# Relationship of the knee extensor strength but not the quadriceps femoris muscularity with sprint performance in sprinters: a reexamination and extension

**DOI:** 10.1186/s13102-021-00293-1

**Published:** 2021-06-10

**Authors:** Miyuki Hori, Tadashi Suga, Masafumi Terada, Takahiro Tanaka, Yuki Kusagawa, Mitsuo Otsuka, Akinori Nagano, Tadao Isaka

**Affiliations:** 1 Faculty of Sport Science, Nippon Sport Science University, Shiga Kusatsu, Japan; 2 Faculty of Sport Science, Nippon Sport Science University, Yokohama, Kanagawa Japan

**Keywords:** Knee extension, Isometric/isokinetic torque, Quadriceps femoris, Muscle volume, Knee extensor moment arm, Magnetic resonance imaging

## Abstract

**Purpose:**

This study examined the relationships of knee extensor strength and quadriceps femoris size with sprint performance in sprinters.

**Methods:**

Fifty-eight male sprinters and 40 body size-matched male non-sprinters participated in this study. The knee extensor isometric and isokinetic strengths were measured using a dynamometer. The isokinetic strength measurements were performed with slow and fast velocities at 60°/s and 180°/s, respectively. The quadriceps femoris muscle volume (MV) was measured using magnetic resonance imaging. The relative knee extensor strengths and quadriceps femoris MV were calculated by normalizing to body mass.

**Results:**

Absolute and relative knee extensor strengths during two velocity isokinetic contractions, but not during isometric contraction, were significantly higher in sprinters than in non-sprinters (*P* = 0.047 to < 0.001 for all). Such a significant difference was also observed for relative quadriceps femoris MV (*P* = 0.018). In sprinters, there were positive correlations between all three knee extensor strengths and quadriceps femoris MV (*r* = 0.421 to 0.531, *P* = 0.001 to < 0.001 for all). The absolute and relative strengths of the fast-velocity isokinetic knee extension correlated negatively with personal best 100-m sprint time (*r* = −0.477 and −0.409, *P* = 0.001 and < 0.001, respectively). In contrast, no such significant correlations were observed between absolute and relative quadriceps femoris MVs and personal best 100-m sprint time.

**Conclusions:**

These findings suggest that despite the presence of the relationship between muscle strength and size, the knee extensor strength may be related to superior sprint performance in sprinters independently of the quadriceps femoris muscularity.

## Background

Superior sprint performance is achieved using gross torques of the lower limb joints [1]. Of the joint torques, the knee extensor joint torque is known to be a major source for performing the rapid and strong knee extension during the swing phase while sprinting [1–3]. Furthermore, the knee extensor torque contributes to accelerating the center of mass of the body and maintaining the height of the center of mass of the body during the stance phase while sprinting [1–3]. These roles of the knee extensor torque help increasing peak vertical ground reaction forces during sprinting [1–3], which is a major determinant of sprint performance [4, 5]. The joint torque can be generally assessed as the strength of the muscle(s) around the joint. Therefore, the knee extensor strength may be an important variable for achieving superior sprint performance in sprinters.

Previous studies have reported that higher knee extensor muscle strength is related to better sprint performance in athletes [6–9], including sprinters [6, 7]. A first study by Alexander et al. [6] determined that higher isokinetic knee extensor strength is correlated with better personal best 100-m sprint time in 14 male sprinters. Following their study, Dowson et al. [7] also reported that higher isokinetic knee extensor strength is correlated with better 15-m sprint time in 24 male athletes, including 8 sprinters. Nevertheless, the two previous studies did not examine the relationship between isometric knee extensor strength and sprint performance in sprinters. A recent study by Miller et al. [10] reported that isometric knee extensor strength did not correlate with 100-m sprint performance in 31 male sprinters, which was a relatively large sample size. These findings suggest that isokinetic strength, rather than isometric strength, of knee extension may be a more important variable for superior sprint performance in sprinters. However, previous studies that examined the relationship between isokinetic knee extensor strength and sprint performance in sprinters were conducted with small sample sizes [6, 7]. Therefore, to clarify our understanding of this relationship, it would be beneficial to reexamine sprinters with large sample size.

The magnitude of muscle strength, including isokinetic strength, of knee extensors is primarily determined by agonist quadriceps femoris size in untrained individuals [11–14]. However, to the best of our knowledge, no study has examined such a relationship in sprinters. Despite this theoretical relationship, previous studies have determined the lack of correlation between quadriceps femoris size and sprint performance in sprinters [15–19]. Based on this finding, it is hypothesized that the knee extensor strength would be related to sprint performance in sprinters independently of the quadriceps femoris muscularity; however, this hypothesis has not been clarified yet. To test this hypothesis, we first compared the knee extensor strength and quadriceps femoris size in sprinters and non-sprinters in order to understand the features of these variables in sprinters. Second, we examined the relationship between knee extensor strength and quadriceps femoris size in sprinters. Third, we examined the relationships of the knee extensor strength and quadriceps femoris size with 100-m sprint performance in sprinters.

## Methods

### Participants

Prior to this study, we calculated *a priori* sample size using the effect sizes obtained in our previous study [20]. To compare morphological variable between sprinters and non-sprinters, the necessary minimum number of participants for each group was 20, as calculated from an effect size of 0.93, α-level of 0.05, and β-level of 0.2 (80% power). Additionally, to determine the relationship between morphological variable and sprint performance in sprinters, the necessary minimum number of sprinters was 16, as calculated from an effect size of 0.56, α-level of 0.05, and β-level of 0.2 (80% power).

Fifty-eight well-trained male sprinters (age: 21 ± 2 years) participated in this study. Their best official records of a 100-m race (i.e., personal best 100-m sprint time) within the previous 1 year were obtained by self-report, and these times were verified on the official homepage of the track and field team of the university or company to which they were affiliated. The personal best 100-m sprint times among sprinters ranged from 10.32 to 11.80 sec (mean, 11.13 ± 0.36 sec). All sprinters were involved in regular sprint training at least 5 times per week and had regularly competition. In addition, 40 non-sprinters (age: 22 ± 2 years) participated as a control group. Their physical characteristics (i.e., body height and body mass) were similar to those of the sprinters (see Table 1). The body size-matched control participants were recreationally active, but were not involved in any specific physical training program within the previous three years. Nevertheless, many of them had participated in recreational sports and/or physical training for 2–3 h per week. All participants were informed of the experimental procedures and risks and provided their written informed consent prior to the participation in the study. The study was performed in accordance with the Declaration of Helsinki and approved by the Ethics Committee of Ritsumeikan University (BKC-IRB-2016-047).

**Table 1:** Physical characteristics, knee extensor strength and quadriceps femoris size variables in sprinters and non-sprinters

	Sprinters	Non-sprinters	*P* value	Cohen’s *d*
Body height, cm	175.2 ± 5.4	174.1 ± 5.5	0.326	0.20
Body mass, kg	65.4 ± 6.1	65.9 ± 7.0	0.709	0.08
Knee extensor strength				
Isometric strength, Nm	272.9 ± 52.0	257.5 ± 47.5	0.140	0.31
Slow-velocity Isokinetic strength, Nm	189.1 ± 38.8	173.8 ± 34.5	0.047	0.41
Fast-velocity Isokinetic strength, Nm	128.0 ± 27.5	112.5 ± 23.2	< 0.001	0.60
Quadriceps femoris size				
Proximal CSA, cm^2^	53.8 ± 7.0	49.3 ± 8.1	< 0.001	0.61
Middle CSA, cm^2^	79.0 ± 8.4	77.5 ± 10.6	0.443	0.19
Distal CSA, cm^2^	58.2 ± 6.5	58.0 ± 9.1	0.923	0.02
MV, cm^3^	2101.5 ± 250.1	2007.4 ± 270.1	0.080	0.36
Knee extensor strength/body mass				
Isometric strength, Nm/kg	4.2 ± 0.6	3.9 ± 0.6	0.050	0.41
Slow-velocity Isokinetic strength, Nm/kg	2.9 ± 0.5	2.6 ± 0.5	0.017	0.50
Fast-velocity Isokinetic strength, Nm/kg	2.0 ± 0.3	1.7 ± 0.3	< 0.001	0.69
Quadriceps femoris size/body mass				
Proximal CSA, cm^2^/kg^2/3^	3.3 ± 0.4	3.0 ± 0.5	0.001	0.68
Middle CSA, cm^2^.kg^2/3^	4.9 ± 0.4	4.8 ± 0.5	0.241	0.26
Distal CSA, cm^2^/kg^2/3^	3.6 ± 0.3	3.6 ± 0.5	0.731	0.08
MV, cm^3^/kg	32.1 ± 2.7	30.5 ± 3.5	0.018	0.53
Knee extensor strength/quadriceps femoris MV				
Isometric strength, kNm/cm^3^	130.2 ± 22.1	128.5 ± 18.3	0.681	0.08
Slow-velocity Isokinetic strength, kNm/cm^3^	90.1 ± 15.7	86.9 ± 14.3	0.299	0.21
Fast-velocity isokinetic strength, kNm/cm^3^	61.1 ± 12.1	56.3 ± 10.3	0.044	0.42

Values are presented as mean ± standard deviation, *CSA* cross-sectional area, *MV* muscle volume. The slow- and fast-velocity isokinetic knee extensor strengths were measured with 60º/s and 180º/s, respectively

### Knee extensor strength

The method for measuring the knee extensor strength has been described in our previous studies [12, 13, 21, 22]. The isometric and isokinetic knee extensor strengths of the right leg were measured using a dynamometer (BIODEX system 3; BIODEX Medical, Shirley, NY, USA). The hip and thigh of participants were securely fixed by seat belts. The hip and knee joint angles were fixed at 100 ° and 90 ° (full extension was at 180 °), respectively. The ankle joint was attached to a bar connected to the force transducer. Isometric strength measurement was performed at a 90º of the knee joint, which was selected according to our and other previous studies [11–14, 21, 22]. Isokinetic strength measurements were performed with slow and fast angular velocities at 60º/s and 180º/s, respectively, through a 90º to 170º range of motion of the knee joint. Both angular velocities employed in the isokinetic strength measurements were selected according to our and other previous studies [13, 23]. The isometric and isokinetic strength measurements were performed with two trials, or more than two trials. The isometric strength trials were performed for 3 sec each with a 1-min rest period. The isokinetic strength trials were performed consecutively with 3 repetitions per each trial. The rest intervals between the trials were set for 1 min. If the difference between the two strength values of each strength measurement was more than 5 % of the highest value, additional trials were performed until this was corrected, as our previous studies [12, 13, 21, 22]. The highest value of the trials for each strength measurement was used for analysis of this study. To minimize the effects of differences in sizes of body and quadriceps femoris muscle among participants, the absolute isometric and isokinetic strengths were normalized to body mass or quadriceps femoris muscle volume (MV). In the reproducibility of the knee extensor strength measurements, intraclass correlation coefficient (ICC) of isometric knee extensor strength on two separate days in 14 healthy young males obtained in our previous study was 0.974 [21], which can be considered as excellent [24]. Additionally, ICCs of slow- and fast-velocity isokinetic knee extensor strengths on two separate days in 13 boys obtained in our previous study was 0.993 and 0.982, respectively [13].

### Quadriceps femoris size

The method for measuring the quadriceps femoris size has been described in our previous studies [12, 13, 15, 19]. The quadriceps femoris size was measured using a 1.5-T magnetic resonance system (Signa HDxt; GE Medical Systems, WI, USA). Participants were placed in a supine position on the scanner bed, with both knees fully extended and both ankles set at the neutral position (i.e., 0°). To measure the quadriceps femoris size, axial T_1−_weighted scans of the thigh of the right leg were acquired with a standard body coil. The axial scans were obtained in successive slices with an inter distance of 10 mm from the inferior aspect of the greater trochanter to the lower edge of the femur with a repetition time of 600 ms, echo time of 7.6 ms, field of view of 480 mm, and matrix size of 512 × 256 pixels.

Analyses of cross-sectional area (CSA) and MV of the quadriceps femoris were conducted using image analysis software (OsiriX Version 5.6, Switzerland). The CSAs of the quadriceps femoris were calculated from three regions at proximal 25% (i.e., proximal CSA), 50% (i.e., middle CSA), and 75% (i.e., distal CSA) along their length. The MV of the quadriceps femoris was calculated by summing the CSAs along their length at intervals of 10 mm. To minimize the effect of difference in body size among participants, absolute quadriceps femoris size CSAs were normalized to body mass to the two-thirds power. The quadriceps femoris size MV was also normalized to body mass. In the reproducibility of the quadriceps femoris size measurements, ICC of the middle quadriceps femoris CSA on two separate days in 14 healthy young males obtained in our previous study was 0.968 [15].

### Statistical analysis

Data are presented as mean ± stander deviation. All data was checked for normality of distribution using the Shapiro-Wilk test, which were considered normally distributed (*P* = 0.079 to 0.976). Comparisons of measured variables between sprinters and non-sprinters were performed using an unpaired *t*-test. The Cohen’s *d* effect size using the pooled standard deviation was calculated to determine the magnitude of the difference in the variable between the two groups [25]. The strength of effect size was interpreted as small (0.20 to 0.49), medium (0.50 to 0.79), and large (> 0.80) [25]. Relationships between knee extensor strength and quadriceps femoris size variables in sprinters and non-sprinters were evaluated using a Pearson’s product moment correlation coefficient. The same statistics was also performed to determine the relationships of knee extensor strength and quadriceps femoris size variables with personal best 100-m sprint time in sprinters. The strength of correlation was interpreted as negligible (0.00 to 0.29), low (0.30 to 0.49), moderate (0.50 to 0.69), high (0.70 to 0.89), and very high (0.90 to 1.00) [26]. Partial correlation analyses were used to adjust the effects of each of six confounding factors, two physical characteristics (i.e., body height and mass) and four absolute quadriceps femoris size variables (i.e., three CSAs and MV), on a correlation between fast-velocity knee extensor strength and personal best 100-m sprint time in sprinters. A stepwise multiple regression analysis was used to determine the predictive variables for the personal best 100-m sprint time of the sprinters. Prior to this analysis, multicollinearity between variables was checked with a correlation of ≥ 0.85. A potential multicollinearity was observed between absolute and relative (i.e., normalized to body mass and/or quadriceps femoris MV) values of some knee extensor strength and quadriceps femoris size variables. Based on these results, the relative values were not included in the analysis. Therefore, a stepwise multiple regression analysis was conducted with nine variables, two physical characteristics (i.e., body height and body mass), three absolute knee extensor strengths, and four absolute quadriceps femoris size variables (i.e., three CSAs and MV). The level of statistical significance was defined at *P* < 0.05. All statistical analyses were conducted using SPSS software (version 19.0; IBM Corp, Armonk, NY, USA).

## Results

Physical characteristics and knee extensor strength and quadriceps femoris size variables in sprinters and non-sprinters are summarized in Table 1. Body height and mass did not differ significantly between sprinters and non-sprinters (*d* = 0.20 and 0.08, respectively; *P* = 0.326 and 0.709, respectively).

With regard to the knee extensor strength, slow- and fast-velocity isokinetic strengths, but not isometric strength, of the knee extension were significantly higher in sprinters than in non-sprinters (*d* = 0.41 and 0.60, *P* = 0.047 and < 0.001, respectively). Moreover, slow- and fast-velocity knee extensor isokinetic strengths relative to body mass were significantly higher in sprinters than in non-sprinters (*d* = 0.50 and 0.69, *P* = 0.017 and < 0.001, respectively). A trend against such significance was also observed for the relative isometric knee extensor strength (*d* = 0.41, *P* = 0.050). Furthermore, fast-velocity isokinetic strength, but not isometric and slow-velocity isokinetic strengths, of the knee extension relative to quadriceps femoris MV was significantly higher in sprinters than in non-sprinters (*d* = 0.42, *P* = 0.044).

With regard to the quadriceps femoris size, proximal CSA, but not middle and distal CSAs, of the quadriceps femoris was significantly greater in sprinters than in non-sprinters (*d* = 0.61, *P* < 0.001). A trend against such significance was also observed for the quadriceps femoris MV (*d* = 0.36, *P* = 0.080). Furthermore, proximal CSA and MV of the quadriceps femoris relative to body mass were significantly greater in sprinters than in non-sprinters (*d* = 0.68 and 0.53, *P* = 0.001 and 0.018, respectively).

Correlation coefficients between knee extensor strength and quadriceps femoris size variables in sprinters and non-sprinters are shown in Table 2. In sprinters, positive correlations were observed between most variables of knee extensor strength and quadriceps femoris size (*r* = 0.339 to 0.531, *P* = 0.009 to < 0.001 for all), excluding a correlation between fast-velocity isokinetic knee extensor strength and proximal quadriceps femoris CSA (*r* = 0.186, *P* = 0.163). Similar trends of the positive correlations between knee extensor strength and quadriceps femoris size variables were observed for non-sprinters (see Table 2).

**Table 2:** Correlation coefficients between knee extensor strength and quadriceps femoris size variables in sprinters and non-sprinters

	Isometric strength		Slow-velocity isokinetic strength		Fast-velocity isokinetic strength	
	*r*	*P* value	*r*	*P* value	*r*	*P* value
***Sprinters***						
Proximal CSA	0.340	0.009	0.339	0.009	0.186	0.163
Middle CSA	0.450	< 0.001	0.523	< 0.001	0.518	< 0.001
Distal CSA	0.381	0.003	0.463	< 0.001	0.451	< 0.001
MV	0.491	< 0.001	0.531	< 0.001	0.421	0.001
***Non-sprinters***						
Proximal CSA	0.290	0.070	0.193	0.234	0.196	0.225
Middle CSA	0.577	< 0.001	0.549	< 0.001	0.515	0.001
Distal CSA	0.585	< 0.001	0.468	0.002	0.348	0.028
MV	0.637	< 0.001	0.520	0.001	0.453	0.003

*CSA* cross-sectional area, *MV* muscle volume

Correlation coefficients of knee extensor strength and quadriceps femoris size variables with personal best 100-m sprint time in sprinters are shown in Table 3. Fast-velocity isokinetic knee extensor strength correlated negatively with personal best 100-m sprint time (*r* = −0.477, *P* < 0.001). Moreover, fast-velocity isokinetic knee extension strengths relative to body mass and quadriceps femoris MV correlated negatively with personal best 100-m sprint time (*r* = −0.409 and −0.441, respectively, *P* = 0.001 for both). A trend against such a significant negative correlation was also observed between absolute slow-velocity knee extensor strength and personal best 100-m sprint time (*r* = −0.236, *P* = 0.075). In contrast, other absolute and relative strengths of isometric and slow-velocity isokinetic knee extension did not correlate with personal best 100-m sprint time (*r* = −0.129 to 0.083, *P* = 0.571 to 0.335 for all). Additionally, although absolute middle quadriceps femoris CSA showed a trend against significant negative correlation with personal best 100-m sprint time (*r* = −0.226, *P* = 0.088), all other quadriceps femoris size variables did not correlate with personal best 100-m sprint time (*r* = −0.160 to 0.164, *P* = 0.956 to 0.219 for all).

**Table 3:** Correlation coefficients of knee extensor strength and quadriceps femoris size variables with personal best 100-m sprint time in sprinters

	*r*	*P* value
Knee extensor strength		
Isometric strength	−0.076	0.571
Slow-velocity isokinetic strength	−0.236	0.075
Fast-velocity isokinetic strength	−0.477	< 0.001
Quadriceps femoris size		
Proximal CSA	0.010	0.942
Middle CSA	−0.226	0.088
Distal CSA	−0.160	0.230
MV	−0.118	0.378
Knee extensor strength/body mass		
Isometric strength	0.083	0.534
Slow-velocity isokinetic strength	−0.129	0.335
Fast-velocity isokinetic strength	−0.409	0.001
Quadriceps femoris size/body mass		
Proximal CSA	0.164	0.219
Middle CSA	−0.065	0.629
Distal CSA	0.007	0.956
MV	0.162	0.224
Knee extensor strength/quadriceps femoris MV		
Isometric strength	−0.007	0.960
Slow-velocity isokinetic strength	−0.186	0.161
Fast-velocity isokinetic strength	−0.441	0.001

*CSA* cross-sectional area, *MV* muscle volume

Partial correlation coefficients between fast-velocity knee extensor strength and personal best 100-m sprint time in sprinters are shown in Table 4. The partial correlation analyses revealed that a negative correlation between absolute fast-velocity isokinetic knee extensor strength and personal best 100-m sprint time remained significant after adjusting for each of physical characteristics and quadriceps femoris size variables (partial *r* = −0.394 to −0.519, *P* = 0.002 to < 0.001 for all).

**Table 4:** Partial correlation coefficients between fast-velocity knee extensor strength and personal best 100-m sprint time in sprinters

Confounding variables	Partial *r*	*P* value
Body height	−0.519	< 0.001
Body mass	−0.397	0.002
Proximal quadriceps femoris CSA	−0.487	< 0.001
Middle quadriceps femoris CSA	−0.432	0.001
Distal quadriceps femoris size CSA	−0.460	< 0.001
Quadriceps femoris MV	−0.475	< 0.001

*CSA* cross-sectional area, *MV* muscle volume

The result of a stepwise multiple regression analysis for predicting personal best 100-m sprint time in sprinters is shown in Table 5. The best-fit model of the stepwise multiple regression analysis was consisted of absolute strengths of slow- and fast-velocity isokinetic knee extensions (adjusted *R*^2^ = 0.271, *P* < 0.001). Of these variables, the fast-velocity isokinetic knee extensor strength were selected as the most predictive variable for the personal best 100-m sprint time of sprinters (*β*= −0.842, *P* < 0.001).

**Table 5:** Stepwise multiple regression analysis for predicting personal best 100-m sprint time in sprinters

Model	Independent variable	Multiple regression equation	*R*^2^	Adjusted *R*^2^	*F* value	*β*	*P* value
Model 1	Fast-velocity Isokinetic strength (*X*_1_)	*Y* = 11.929 − 0.006 *X*_1_	0.228	0.214	16.511	−0.477	< 0.001
Model 2	Fast-velocity Isokinetic strength (*X*_1_)	*Y* = 11.753 − 0.011 *X*_1_ + 0.004 *X*_2_	0.296	0.271	11.569	−0.842	< 0.001
	Slow-velocity isokinetic strength (*X*_2_)					0.449	

## Discussion

Prior to this study, the relationship between knee extensor strength and sprint performance with each relation to quadriceps femoris size in sprinters had not been explored. In this study, we determined that higher fast-velocity isokinetic knee extensor strength variables are correlated with better personal best 100-m sprint time in sprinters. In contrast, all quadriceps femoris size variables did not correlate with personal best 100-m sprint time. Moreover, we found that the correlation between absolute fast-velocity isokinetic knee extensor strength and personal best 100-m sprint time in sprinters was independent of the size of the quadriceps femoris. Furthermore, we determined that absolute fast-velocity isokinetic knee extensor strength was the most predictive variable for the personal best 100-m sprint time in sprinters. These findings suggest that the fast-velocity knee extensor strength may be an important variable for superior 100-m sprint performance in sprinters. Therefore, this study is the first to determine the positive relationship between knee extensor strength and sprint performance in sprinters independently of the quadriceps femoris muscularity.

Several previous studies have reported that higher isokinetic knee extensor strength is related to better sprint performance in sprinters [6, 7]; however, prior to the present study, further understanding of this relationship had not progressed in recent years. Alexander et al. [6] reported a negative correlation between fast-velocity isokinetic knee extensor strength (i.e., 230º/s) and personal best 100-m sprint time in 14 male sprinters. Dowson et al. [7] also reported negative correlations between absolute and relative strengths of three velocity isokinetic knee extensions (i.e., 60 to 240º/s) and 15-m sprint time in 24 male athletes, including 8 sprinters. Furthermore, they reported that these correlations were stronger with fast-velocity (i.e., 150 and 240º/s) than with slow-velocity (i.e., 60º/s). These previous findings suggest that isokinetic knee extensor strength, especially during faster-velocity contraction, may be related to better sprint performance in sprinters. In the present study, we determined that absolute and relative knee extensor strengths during fast-velocity isokinetic contraction, but not during isometric and slow-velocity isokinetic contractions, are negatively correlated with better personal best 100-m sprint time in 58 sprinters. Therefore, with a relatively large sample size of sprinters, the present findings corroborate the results in previous studies [6, 7].

Using ultrasonography (US), Kubo et al. [27] reported that absolute muscle thickness (MT) of the anterior thigh (i.e., quadriceps femoris) is negatively correlated with personal best 100-m sprint time in sprinters. Monte and Zamparo [28] also reported that absolute MTs of the four quadriceps femoris muscles are negatively correlated with personal best 100-m sprint time in sprinters. Nevertheless, MRI is known to be a more appropriate apparatus to measure muscle size than US [29–31]. Using MRI, our previous studies reported that absolute and relative (i.e., normalized to body mass) middle CSAs of the quadriceps femoris did not correlate with personal best 100-m sprint time in sprinters [15, 19]. Moreover, Sugisaki et al. [18] analyzed MRI-measured MV, which is the most appropriate marker of muscle size [29, 30], and reported no correlations between absolute and relative quadriceps femoris MVs and personal best 100-m sprint time in sprinters. Similarly, some previous studies have reported that MRI-measured absolute and/or relative quadriceps femoris MVs did not correlate with sprint performance in sprinters [10, 16, 17]. In the present MRI study, we determined that although a trend against significance negative correlation was observed between absolute middle quadriceps femoris CSA and personal best 100-m sprint time, other quadriceps femoris size variables did not correlate with personal best 100-m sprint time in sprinters. Therefore, the present findings corroborate the results of previous studies [15–19]. Altogether, we suggest that greater quadriceps femoris may not be an essential morphological factor for achieving better 100-m sprint performance in sprinters.

Because muscle strength (i.e., joint torque) is theoretically expressed as the product of muscle force and joint moment arm (MA) dimension, the magnitude of the knee extensor strength is determined not only by the quadriceps femoris size but also by the knee extensor MA. Previous studies have reported a positive correlation between muscle strength and MA of knee extensors in untrained individuals [11–14]. Furthermore, our previous study determined that the knee extensor MA is negatively correlated with personal best 100-m sprint time in sprinters [15]. This may be due to the potential relationship between knee extensor strength and MA; however, no study has examined this relationship in sprinters. If this relationship is observed for sprinters, it may help our understanding of the present findings that higher knee extensor strength is related to better 100-m sprint performance in sprinters independently of the quadriceps femoris size.

As another explanation in terms of morphological factors, the muscle fascicle length may be involved in determining muscle strength [11, 14, 32]. Blazevich et al. [11] reported that the fascicle length of the quadriceps femoris muscle (i.e., vastus lateralis) was a positive predictive variable for the fast-velocity isokinetic strength, but not the isometric and slow-velocity isokinetic strengths, of knee extensors in untrained individuals. Additionally, previous studies have reported that the fascicle length of the quadriceps vastus lateralis is negatively correlated with better 100-m sprint performance in sprinters [33, 34]. Therefore, in addition to the knee extensor MA, muscle fiber fascicle length of the quadriceps femoris may also be an important morphological factor that contributes to our understanding of the present findings, especially by showing the relationship between fast-velocity isokinetic knee extensor strength and 100-m sprint performance in sprinters.

In terms of physiological factors, the muscle fiber composition is known to be an important factor for determining muscle strength [35–37]. Previous studies have reported that the fast-twitch fiber composition of the quadriceps vastus lateralis is positively correlated with isokinetic knee extensor strength [35–37]. In particular, this relationship is observed for faster-velocity contraction more than for slower-velocity contraction of knee extension [35–37]. Furthermore, previous studies have reported that the fast-twitch fiber composition of the quadriceps vastus lateralis is positively correlated with sprint performance in athletes, including sprinters [38, 39]. Therefore, in addition to the morphological factors, these previous findings may help our understanding of the present findings.

The results of this study showed that absolute strengths of the two velocity isokinetic knee extensions were higher in sprinters than in body size-matched non-sprinters, whereas no such a difference was observed for absolute isometric knee extensor strength. Similar results were also observed for the relative knee extensor strengths normalized to body mass. In a previous study, Miller et al. [10] reported that although absolute and relative isometric strengths of knee extension were higher in sub-elite sprinters than in untrained individuals, these strength variables did not differ between elite sprinters and untrained individuals. Their findings suggest that higher isometric knee extensor strength may not be required for successful sprinters. Thus, sprinters may be specifically characterized by a higher isokinetic strength, but not isometric strength, of knee extensors. Additionally, in the present study, we found that effect sizes of the differences in the two velocity isokinetic knee extensor strengths between sprinters and non-sprinters were relatively larger with fast-velocity than with slow-velocity (i.e., 0.60 [medium] and 0.41 [small], respectively), indicating that higher isokinetic knee extensor strength of sprinters may be conspicuous for fast-velocity contraction than for slow-velocity contraction. These findings may be a natural adaptation because sprint training is performed with fast dynamic movements. In addition to this reason, previous studies have reported that the knee extensor MA dimension and quadriceps femoris fascicle length were longer in sprinters than in non-sprinters [15, 33, 34]. Moreover, the fast-twitch fiber composition of the quadriceps femoris is higher in sprinters than in non-sprinters [40, 41]. Furthermore, the magnitude of neural activity (i.e., electromyographic activity) during muscle contraction is known to be a major determinant of muscle strength [14, 42]. Indeed, previous studies have reported that neural activity level during isokinetic contraction may be higher in sprinters than in non-sprinters [43, 44]. These findings may contribute to our understanding of the difference in isokinetic knee extensor strength, especially during fast-velocity contraction, between sprinters and non-sprinters.

Kubo et al. [27] reported that the absolute quadriceps femoris MT did not differ between sprinters and body size-matched non-sprinters. Our previous study also reported that no difference for the absolute and relative middle quadriceps femoris CSAs between sprinters and body size-matched non-sprinters [15, 19]. In contrast, the present study observed a trend against significance with a greater absolute quadriceps femoris MV in sprinters than body size-matched non-sprinters. Furthermore, the relative quadriceps femoris MV normalized to body mass was greater in sprinters than in non-sprinters, which corroborates the result of a study by Miller et al. [10]. These present results might be mainly because of a greater proximal quadriceps femoris CSA in sprinters than non-sprinters. Abe et al. [45] reported that MTs at the proximal and middle regions of the quadriceps femoris were greater in sprinters than in non-sprinters; however, body mass was heavier in sprinters than in non-sprinters. Several previous studies have reported that absolute and relative MVs of the rectus femoris, but not of other three quadriceps femoris muscles, were greater in sprinters than in body size-matched non-sprinters [17, 46]. Composition of the rectus femoris CSA relative to a total of the four quadriceps femoris CSAs is higher at the proximal region than at the middle and distal regions [47]. Therefore, greater MV and proximal CSA of the quadriceps femoris in sprinters than non-sprinters observed in this study may be attributed to a specific hypertrophy of the rectus femoris among the quadriceps femoris n sprinters.

This study has some limitations. In the present study, body mass was used mainly as a variable for normalizing the knee extensor strength and quadriceps femoris size variables. It is well known that sprinters have a lower body fat percentage and whole-body fat mass than non-sprinters [33, 48]. Thus, although we observed that body mass did not differ between the two groups, whole-body muscle mass might be greater in sprinters than in non-sprinters. This possibility may be partially indicated by the present result that the quadriceps femoris MV relative to body mass was greater in sprinters than in non-sprinters. Considering these findings, to compare the knee extensor strength and quadriceps femoris size variables between the two groups, it would be more appropriate to normalize these variables with whole-body muscle mass than with body mass. As an alternative to this concern, this study attempted to normalize the knee extensor strengths with quadriceps femoris MV as a regional muscle mass, which can be considered as the underlying strength of the knee extensors [12, 13]. In the results, the relative knee extensor strength during fast-velocity isokinetic contraction, but not during isometric and slow-velocity contractions, was greater in sprinters than in non-sprinters. This finding can be established the fact of a higher fast-velocity isokinetic knee extensor strength in sprinters than non-sprinters. Nevertheless, Abe et al. [49] reported that body fat percentage is positively correlated with personal best 100-m sprint time in sprinters. Furthermore, a series of studies by Abe et al. [33, 49–51] suggested that a lower body fat percentage is associated with better sprint performance in sprinters. Based on their findings, body fat percentage and/or whole-body fat mass would be required to clarify the relationship between fast-velocity knee extensor strength and personal best 100-m sprint time in sprinters. Therefore, the lack of body composition measurement in the present study is a major limitation.

In addition to the lack of body composition measurement, we suggested that the results of this study are affected by other morphological (i.e., joint MA dimension and muscle fascicle length), physiological (i.e., fast-twitch fiber composition), and neuromuscular factors. Thus, the lack of measurements related to these factors is also considered a limitation of this study. Furthermore, we did not measure biomechanical data during 100-m sprinting in sprinters. Thus, we cannot explain in detail the potential impact of the knee extensor strength on 100-m sprint performance. Nevertheless, the higher knee extensor strength appears to help increasing peak vertical ground reaction force during the stance phase while 100-m sprinting [1–3]. The increased peak vertical ground reaction force contributes to shortening contact time and increasing step frequency [1, 4]. These kinetic and kinematic variables are important biomechanical determinants for superior 100-m sprint performance [4, 5]. Although the present study determined significant correlations between fast-velocity knee extensor strength variables and 100-m sprint performance in sprinters, these correlations were low (i.e., *r* = −0.409 to −0.477). Because superior sprint performance is determined by various factors, further studies are needed to examine the effects of some factors underlying the relationships between fast-velocity knee extensor strength and sprint performance in sprinters.

As another limitation of this study, we did not measure sprint performance in non-sprinters. This measurement would be useful to understand the essential importance of knee extensor strength on sprint performance in various populations. Previous studies have reported a positive relationship between knee extensor strength and sprint performance in young term sport athletes [8, 9]. To the best of our knowledge, no study has examined this relationship in untrained individuals. Nevertheless, previous studies have reported that long-term resistance training increases sprint performance, potentially by enhancing the knee extensor strength, in recreationally active young individuals [52, 53]. This population was the same population to non-sprinters recruited in this study. Therefore, it is hypothesized that higher knee extensor strength would play an important role in achieving better sprint performance in various populations, including untrained population. Nevertheless, to test this hypothesis, further studies are needed to examine the relationship between knee extensor strength and sprint performance in untrained individuals.

## Conclusion

This study demonstrated that despite the presence of the relationship between knee extensor strength and quadriceps femoris size, higher fast-velocity isokinetic knee extensor strength is correlated with better personal best 100-m sprint time in sprinters, whereas no such a correlations was observed for the quadriceps femoris size. These findings suggest that the knee extensor strength, but not the quadriceps femoris muscularity, may be an important factor of superior 100-m sprint performance in sprinters.

## Data Availability

The datasets used and/or analyzed during this study are not publicly available because the authors do not have permission from the participants to publicly share their individual data, but are available from the corresponding author on reasonable request.

## Abbreviations

CSA: Cross-sectional area
MA: Moment arm
MRI: Magnetic resonance imaging
MT: Muscle thickness
MV: Muscle volume
US: Ultrasonography
