# Correction to: Relationship of the knee extensor strength but not the quadriceps femoris muscularity with sprint performance in sprinters: a reexamination and extension

**DOI:** 10.1186/s13102-021-00304-1

**Published:** 2021-07-09

**Authors:** Miyuki Hori, Tadashi Suga, Masafumi Terada, Takahiro Tanaka, Yuki Kusagawa, Mitsuo Otsuka, Akinori Nagano, Tadao Isaka

**Affiliations:** 1 Faculty of Sport and Health Science, Ritsumeikan University, Kusatsu, Shiga Japan; 2 Faculty of Sport Science, Nippon Sport Science University, Yokohama, Kanagawa Japan

**Correction to: BMC Sports Sci Med Rehabil 13, 67 (2021)**

**https://doi.org/10.1186/s13102-021-00293-1**

Following publication of the original article [1], the authors reported an error in the affiliations of their article. The correct affiliations are:

1. Faculty of Sport and Health Science, Ritsumeikan University, Kusatsu, Shiga, Japan.

2. Faculty of Sport Science, Nippon Sport Science University, Yokohama, Kanagawa, Japan.

The original article [1] has been corrected.
